# DOK3 is involved in microglial cell activation in neuropathic pain by interacting with GPR84

**DOI:** 10.18632/aging.202144

**Published:** 2020-12-03

**Authors:** Wen-Shuang Gao, Yu-Juan Qu, Juan Huai, Hui Wei, Yang Zhang, Shou-Wei Yue

**Affiliations:** 1 Rehabilitation Center, Qilu Hospital, Cheeloo College of Medicine, Shandong University, Jinan, China

**Keywords:** DOK3, microglia, neuropathic pain, GPR84, pregabalin

## Abstract

Adaptor molecule downstream of kinase-3 (DOK3) is a vital regulator of innate immune responses in macrophages and B cells, and G-protein-coupled receptor 84 (GPR84) is significant in mediating the biosynthesis and maintenance of inflammatory mediators that are induced by neuropathic pain in microglia. In the present study, we determined the role of DOK3 in activating microglia-induced neuropathic pain and investigated the underlying mechanisms associated with GPR84. We found that knockdown of DOK3 in microglial cells dramatically reduced the levels of inflammatory factors, and we uncovered a physical association between DOK3 and GPR84 in the induction of inflammatory responses. We also observed that neuropathic pain and inflammatory responses induced by chronic constriction injury (CCI) of the sciatic nerve or intrathecal injection of a GPR84 agonist were compromised in DOK3^-/-^ mice *in vivo*. Finally, enforced expression of DOK3 provoked inflammatory responses, and administration of pregabalin relieved neuropathic pain via inhibition of DOK3 expression. In conclusion, DOK3 induced neuropathic pain in mice by interacting with GPR84 in microglia. We hypothesize that targeting the adaptor protein DOK3 may open new avenues for pharmaceutical approaches to the alleviation of neuropathic pain in the spinal cord.

## INTRODUCTION

Neuropathic pain—characterized by spontaneous pain and hyperalgesia to normally innocuous stimuli—is a refractory clinical problem and reflects aberrant excitability of the nervous system, with multiple anatomical and functional alterations following peripheral nerve injury [1, 2]. In the central nervous system (CNS), microglial cells are regarded as resident macrophages that originate from primitive macrophages of the yolk sac [3]. These cells are widely distributed throughout the CNS and monitor the local environment to allow a rapid response to threats from a wide range of stimuli [4]. In the case of neuropathic pain, microglial molecules vary with modulations in the IFN-γ/IFN-γ R system [5]; changes in toll-like receptors 2, 3, or 4 [6, 7]; upregulation of purinergic P2X4 receptors [8]; and modifications of intracellular signaling molecules (mitogen-activated protein kinases [MAPKs]) [9, 10]. In addition, microglia play a crucial role in the interaction with neighboring neurons in the development of pain hypersensitivity [11, 12]. Therefore, spinal microglia constitute a promising target in reversing disordered functioning of the nervous system [13], and it is thus essential to uncover the mechanisms of pain hypersensitivity to better understand microglial molecular functions and develop new approaches for neuropathic pain.

As molecular scaffolds, adapter proteins precisely organize positive and negative effectors into signaling complexes. A typical adaptor molecule is the DOK3 (downstream of kinase-3) protein, which is preferentially expressed in immune cells such as macrophages and B cells [14]. To the best of our knowledge, DOK3 has been principally implicated in negative-feedback regulation, and involved in facilitating or sustaining the activation of inhibitory molecules in B cells [15–17]. As reported previously, overexpression of DOK3 increased the anti-inflammatory effects of vitamin B6 in macrophages [18], and degradation of DOK3 in TLR9 signaling increased the production of IL-6 and TNF-α in macrophages [19].

Intriguingly, there is still controversy as to whether the primary function of some adapters is to exert positive or negative effects. With respect to synapse-associated proteins (SAP) [20], a facilitative or inhibitory role depends upon cell type or condition. Investigators have reported that the adapter protein DOK3 plays an essential role in the evolution of B cells, and that the generation of plasma cells (PCs) was significantly attenuated in DOK3^-/-^ mice [21]. In addition, upon viral influenza infection, the synthesis of IFN-β was impaired in DOK3-deficient macrophages. DOK3 also plays a critical role in TLR3 signaling in macrophages [22]. Collectively, these results are credible, although apparently contradictory in theory; and further work is thus required to provide reasonable explanations for the seemingly incongruous effects of the adapter molecule DOK3.

As a membrane-bound receptor, GPR84 is markedly upregulated in macrophages and microglia in mediating the production and maintenance of inflammatory mediators in neuropathic pain [23], and in mice suffering from endotoxemia or multiple sclerosis, microglia express GPR84 in a strong and sustained manner [24]. GPR84 was also a potent target in the treatment of inflammation-associated disease [25].

Understanding mechanisms that govern the inhibition of inflammatory responses is important for treating neuroinflammation that can give rise to serious issues of neuropathic pain. Although microglia originate from macrophages, the role of DOK3 in microglia remains unclear presently, and there is a paucity of evidence regarding the association between DOK3 and GPR84 proteins. Based upon the above analysis, we speculated that DOK3 and GPR84 would interact in pain and inflammatory diseases in microglia, potentially providing novel insights into the treatment of neuropathic pain.

## RESULTS

### Knockdown of DOK3 contributes to a reduction in inflammatory factors in microglia

**Figure 1:**
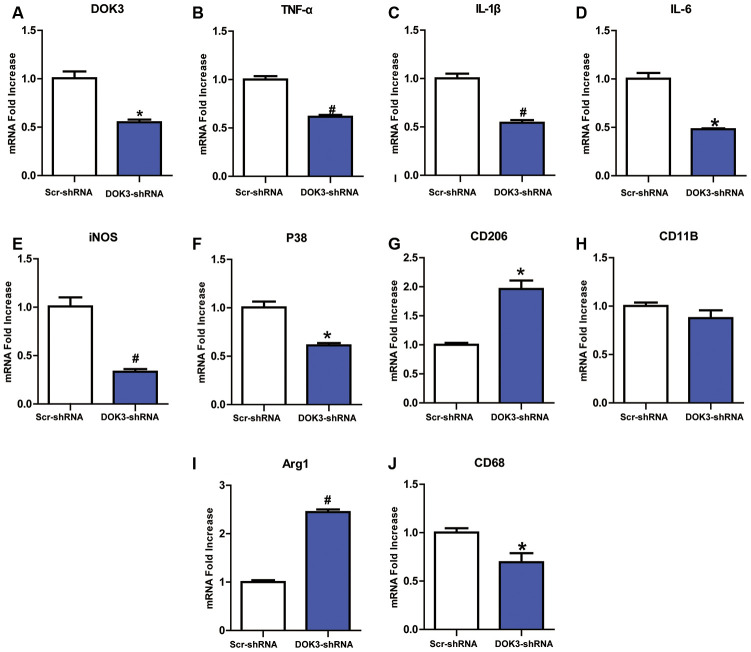
**Downregulation of DOK3 reduces the levels of inflammatory cytokines in microglia.** Microglia were infected with a DOK3-specific short hairpin RNA (shRNA) to establish stably down-regulated DOK3 expression. Total cell lysates of DOK3-shRNA and Scr-shRNA microglia were used to determine the levels of mRNA for DOK3 (**A**), TNF-α (**B**), IL-1β (**C**), IL-6 (**D**), iNOS (**E**), P38 (**F**), CD68 (**G**), CD11B (**H**), Arg1 (**I**), and CD206 (**J**) by quantitative reverse transcription-polymerase chain reaction (RT-qPCR). N=3 per group, and data are presented as means ± SEM. *p < 0.05, #*p <* 0.01 vs. Scr-shRNA.

DOK3 appears to be involved in immune defense by macrophages and B cells [15, 19], but its role in microglia remains underappreciated. To first determine a role for DOK3 in microglia, we infected microglia with a lentivirus containing a DOK3-specific short hairpin RNA (shRNA) to elucidate whether down-regulated DOK3 expression altered microglial inflammatory responses. As shown in Figure 1A, DOK3 mRNA levels decreased by almost 50% in DOK3-shRNA cells compared with vehicle-treated microglia (Scr-shRNA), and protein levels were confirmed by western blotting analysis (Supplementary Figure 1D). Several inflammatory mediators associated with nociceptive or inflammatory signaling showed a greater down-regulation in DOK3-shRNA than in Scr-shRNA microglia, including TNF-α (Scr-shRNA 1.0±0.06 vs. DOK3-shRNA 0.62±0.03, *p <* 0.01), IL-1β (Scr-shRNA 1.0±0.08 vs. DOK3-shRNA 0.54±0.05, *p <* 0.01), IL-6 (Scr-shRNA 1.0±0.06 vs. DOK3-shRNA 0.88±0.14, *p <* 0.05), P38 (Scr-shRNA 1.0±0.11 vs. DOK3-shRNA 0.61±0.04, *p <* 0.05), iNOS (Scr-shRNA 1.0±0.16 vs. DOK3-shRNA 0.33±0.05, *p <* 0.01), and CD68 (Scr-shRNA 1.0±0.07 vs. DOK3-shRNA 0.69±0.16, *p <* 0.05, Figure 1B–1G). These molecules are also important inflammatory mediators during microglial activation. Conversely, Arg1 and CD206 (as typical M2 markers employed by M2 microglia to antagonize inflammatory responses [26, 27]) were induced to a greater degree in the DOK3-shRNA microglia (Scr-shRNA 1.0±0.06 vs. DOK3-shRNA 2.45±0.09, *p <* 0.01; Scr-shRNA 1.0±0.06 vs. DOK3-shRNA 1.96±0.26, *p <* 0.05, Figure 1I, 1J). The levels of CD11B mRNA were only slightly changed, with no significant difference between the 2 groups. These findings suggested that DOK3 regulated the release of a subset of inflammatory mediators in microglia under physiologic conditions, and that it is therefore plausible that the DOK3-shRNA phenotype was responsible for the reduced capacity of microglia to launch an inflammatory response.

It is well known that GPR84 is restricted to microglia in an unstimulated state and strongly up-regulated by LPS, which suggests a role for GPR84 in the regulation of microglia and neuroinflammatory processes [25, 28]. Thus, based on preliminary experiments, we proposed a mechanism by which DOK3 might directly interact with GRP84 in microglia, and it became worthwhile exploring the function of DOK3 in GPR84-mediated pathologic processes in greater depth.

### GPR84 induces inflammatory responses via DOK3

**Figure 2:**
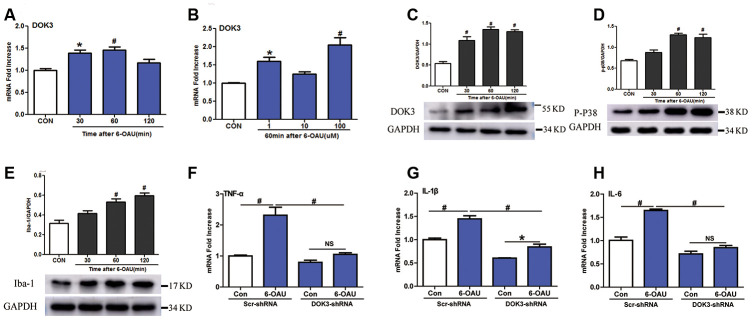
**GPR84 activation-induced inflammatory responses are partially mediated via DOK3 in microglia.** Microglia were incubated with 6-n-octylaminouracil (6-OAU) for 120 min at different concentrations (1, 10, or 100 μM). (**A**, **B**) DOK3 mRNA levels at different time-points were determined by RT-qPCR. (**C**–**E**) DOK3 (**C**), p-p38 (**D**), and Iba-1 (**E**) protein levels were determined using western immunoblotting analysis. (**F**–**H**) Lentivirus-infected microglia were exposed to 6-OAU (1 μM, 60 min); and TNF-α (**F**), IL-1β (**G**), and IL-6 (**H**) mRNA levels were determined by RT-qPCR. N=3 per group, data are presented as means ± SEM. *p < 0.05, #*p <* 0.01, NS, not significant for comparisons vs. control (CON) or between the 2 groups connected by the horizontal line.

Knowing that GPR84 activation promoted a proinflammatory microglial phenotype [24], we next determined whether an activated GPR84-increased inflammatory response in microglia was due to a direct effect of DOK3. We incubated microglia with 6-n- octylaminouracil (6-OAU, a surrogate agonist for GPR84) for different durations (30, 60, and 120 min) and at different concentrations (1, 10, or 100 μM). As depicted in Figure 2A–2C, 6-OAU in a time-dependent fashion increased DOK3 mRNA and protein at a concentration of 1 μM and increased DOK3 in a concentration-dependent fashion after 60 min of exposure. Since p-p38 and ionized calcium-binding adapter molecule-1 (Iba-1) are key kinases in nociceptive pathways and correlate with microglial activation [23, 24], we also assessed whether GPR84 activation modulated p-p38 and Iba-1 levels. As we expected, there was an equivalent increase in both p-p38 and Iba-1 protein expression as DOK3 protein was upregulated after 6-OAU treatment (Figures 2D, 1E), and this was commensurate with increased levels of IL-1β, TNF-α, and IL-6 (Supplementary Figure 1A–1C). Collectively, these data suggested that the increased p-p38 and Iba-1 protein levels—accompanied by increased TNF-α, IL-6, and IL-1β mRNA levels—were associated with increased DOK3 in microglia.

To test this hypothesis, we ascertained whether down-regulated DOK3 expression in microglia was immunosuppressed by inhibiting the release of TNF-α, IL-1β, or IL-6 under GPR84-activated conditions. As shown in Figure 2F–2H, after treatment with 6-OAU (1 μM, 60 min), the capacity to synthesize the inflammatory mediators TNF-α (F), IL-1β (G), and IL-6 (H) was dramatically impaired under GPR84-activated conditions in DOK3-shRNA microglia compared with the Scr-shRNA group: TNF-α, Scr-shRNA, 2.3-fold vs. DOK3-shRNA, 1.25-fold); IL-1β, (Scr-shRNA, 1.45-fold vs. DOK3-shRNA, 1.4-fold; and IL-6, Scr-shRNA, 1.65-fold vs. DOK3-shRNA, 1.2-fold). Furthermore, 6-OAU increased DOK3 mRNA levels, but there was no significant change in GPR84 protein expression in the 2 groups (Supplementary Figure 1E). We speculate that DOK3—as a downstream target of the GPR84-signaling pathway—is an important component in immune regulation by microglia. Our findings support the premise that DOK3 is essential for up-regulating TNF-α, IL-1β, and IL-6 expression in microglia so as to promote inflammatory responses under physiologic or pathologic conditions. In its absence, the response of microglia cells to inflammatory insult was significantly impaired.

Previous studies have shown that under certain conditions DOK3 translocates to the plasma membrane to recruit or integrate signaling molecules into various signaling pathways by a physical association with them, including DAP12 or Grb2 [29–31]. Thus, it was important to address the manner in which DOK3 and GPR84 interacted in microglia.

### Incubation of microglia with LPS or GPR84 agonist induces a physical association between DOK3 and GPR84

**Figure 3:**
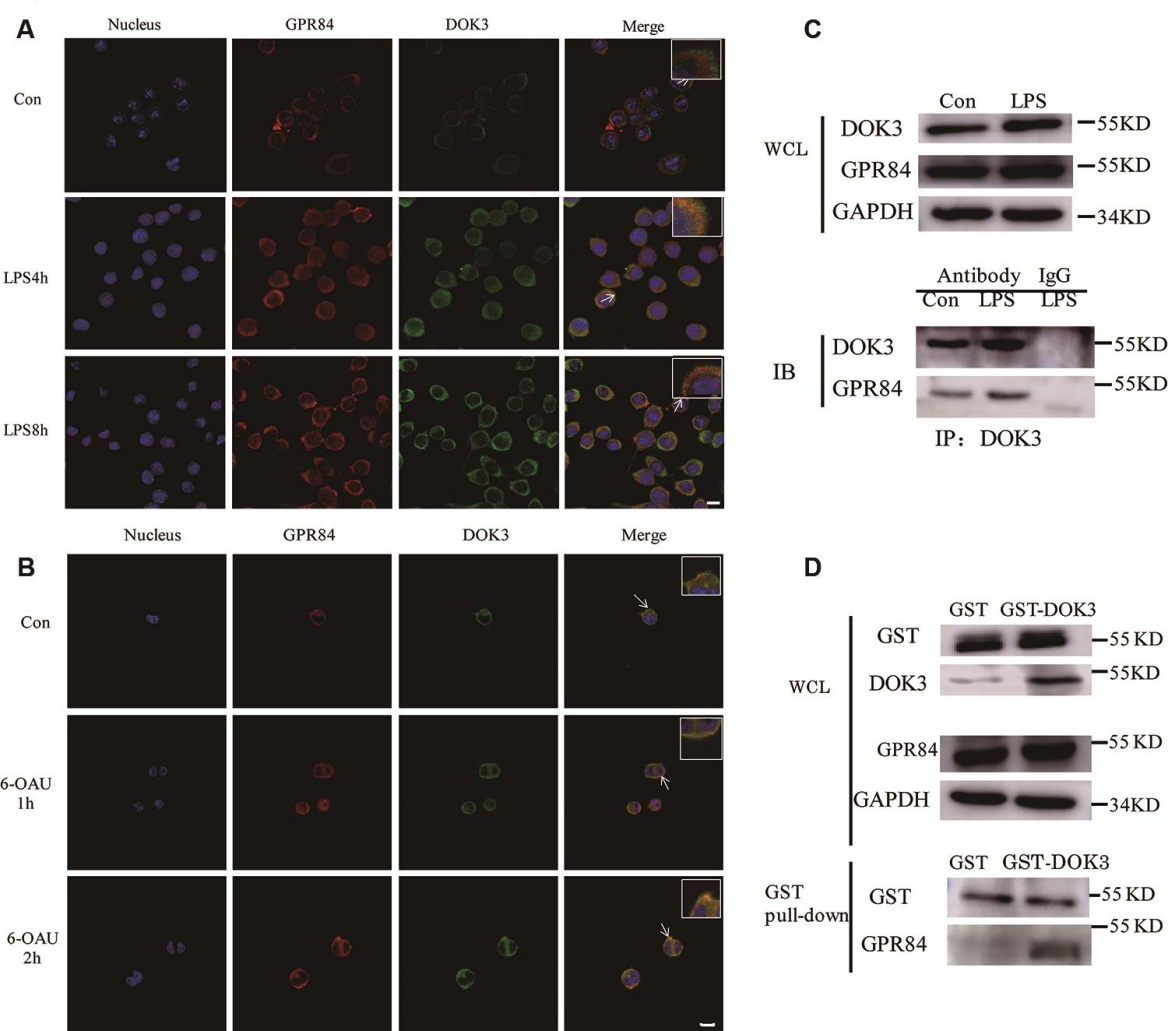
**GPR84 proteins are physically associated with DOK3 in microglia.** (**A**) Microglia were pretreated with LPS (1 μM) for 4 or 8 h, and the levels of GPR84 and DOK3 protein were assayed by immunofluorescence analysis. (**B**) Microglia were pretreated with 6-OAU (1μ M) for 1 or 2 h, and the levels of GPR84 and DOK3 protein were assayed by immunofluorescence analysis. Scale bar, 10 μm. (**C**) Microglia were pretreated with LPS (1 μM) for 4 h, and the levels of GPR84 in total cell lysates were determined by co-immunoprecipitation and western blotting. (**D**) Purified recombinant human GST or GST-DOK3 proteins were incubated with microglia for 2 h in reaction buffers. Total cell lysates or reaction products were then used in GST-pulldown assays and western blotting to determine the levels of GPR84.

Many adapters are indispensable to the development and functioning of immune cells. Our results showed that LPS stimulated the translocation of DOK3 from the cytosol to the plasma membrane in microglia (Supplementary Figure 2A), consistent with other reports [31, 17] that demonstrated that DOK3 functioned predominantly in the plasmalemma. Moreover, incubation with LPS attenuated microglial cell viability (Supplementary Figure 2B) and rapidly increased TNF-α, IL-6, and IL-1β mRNA levels and decreased Arg1 expression (Supplementary Figure 2C–2F). After incubation with LPS, microglia were thereby rapidly transformed into a M1 phenotype. To investigate the correlation between GPR84 and DOK3 in microglia, we incubated microglia with LPS (1 μM) for different durations (0, 4, and 8 h), and we demonstrated that the fluorescence intensity of GPR84 and DOK3 in the plasma membrane was significantly enhanced after LPS treatment compared with the control group (Figure 3A). Furthermore, GPR84 and DOK3 were readily co-localized in microglia, and the merged fluorescence was progressively increased with LPS incubation.

To further investigate whether GPR84 functioned by a physical association with DOK3, we incubated microglia with 6-OAU (1 μM) for 1 and 2 h, using the same volume of DMSO as the control group. Immunofluorescence (IFC) analysis indicated that the levels of DOK3 and merged fluorescence were higher in the plasma membrane after 6-OAU treatment, indicating that GPR84 functions by physically associating with DOK3 (Figure 3B).

Considering the co-localization phenomenon of DOK3 and GPR84 in microglia, we speculated that DOK3 and GPR84 constituted a signaling complex that could induce downstream inflammatory signals. To investigate this hypothesis, we performed co-immunoprecipitation (IP) analyses of whole microglial cell lysates (WCL). We stimulated microglial cells with LPS (1 μM) for 4 h, lysed the cells, and then subjected them to immunoprecipitation with an anti-DOK3 antibody and immunoblotted them with an anti-GPR84 antibody. As shown in Figure 3C, western blotting analysis showed that GPR84 was physically associated with DOK3 under either physiologic or LPS-stimulated conditions. Furthermore, to avoid the influence of anti-DOK3 antibodies added to the reactants, we generated a glutathione S-transferase (GST)-DOK3 fusion protein for pull-down assays, with homozygous GST proteins used as a control. Purified recombinant GST-DOK3 fusion protein was incubated with WCL *in vitro*, and we showed that—consistent with the IP results—GST-DOK3 pulled down GPR84 from lysates of microglia stimulated by LPS (1 μM), whereas the GST control protein did not (Figure 3D). These results further confirmed the manner in which DOK3 and GPR84 interacted in microglia, and provided a theoretical basis for the future targeted therapy of pain and inflammatory diseases.

### CCI-induced neuropathic pain and inflammatory responses are alleviated in DOK3^-/-^ mice

It was previously demonstrated that the model of CCI-induced pain hypersensitivity effectively and rapidly produced a persistent mechanical allodynia, which was maintained for at least 3 weeks [32, 33].

**Figure 4:**
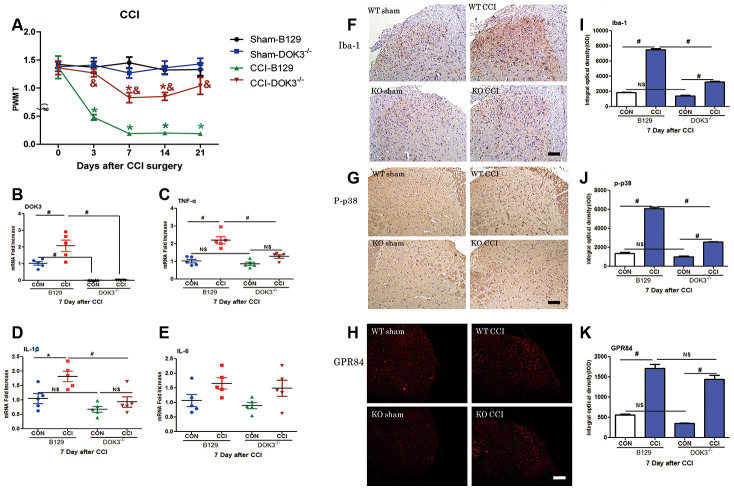
**CCI-induced neuropathic pain and inflammatory responses are partially inhibited in DOK3^-/-^ mice.** To induce neuropathic pain, B129 and DOK3^-/-^ mice underwent surgery for chronic constriction of the sciatic nerve to establish the CCI model. (**A**) CCI-induced mechanical allodynia was determined by calculating PWMT on days 0, 3, 7, 14, and 21 after CCI surgery. N=8-10, data are presented as means ± SEM. *p < 0.05 vs. sham group, & *p* < 0.05 vs. WT plus CCI at the same time. (**B**–**E**) Homogenates of lumbar spinal cord isolated from mice were used to determine the levels of mRNA for DOK3 (**B**), TNF-α (**C**), IL-1β (**D**), and IL-6 (**E**) on day 7 after CCI. (**F**–**K**) Iba-1 (**F**), P-p38 (**G**), and GPR84 (H) in lumbar spinal cords of mice were assayed by immunofluorescence and immunohistochemical analysis. Quantities of Iba-1 (**I**), P-p38 (**J**), and GPR84 (**K**) were determined by calculating the integral optical density (IOD). N=8-10, *p < 0.05; #*p* < 0.01; NS, not significant when comparing the 2 groups connected by the horizontal line; scale bar, 50 μm.

To further assess the role of DOK3 in CCI-induced neuropathic pain in the spinal cord, we next examined mechanical thresholds of WT and DOK3^-/-^ mice after CCI surgery. As shown in Figure 4A, compared with the WT controls, the PWMT was significantly decreased from 3 to 21 days postoperatively, demonstrating successful establishment of the CCI model. Although DOK3^-/-^ mice exhibited normal mechanical pain thresholds equivalent to those of WT control mice; after CCI surgery, DOK3^-/-^ mice showed attenuated allodynia compared with the WT mice. These data suggested that under normal physiologic conditions, there was no difference in mechanical pain thresholds between DOK3^-/-^ and WT mice. However, after CCI surgery, the development and persistence of mechanical allodynia were dramatically relieved in DOK3^-/-^ mice. We also found similar changes in the thermal paw withdrawal latency (Supplementary Figure 3A).

Knowing that DOK3 was a valid target in inflammatory responses *in vitro*, we asked whether DOK3 exerted a similar effect *in vivo*. Thus, 7 days after CCI, we harvested the lumbar spinal cords for the following experiments. As depicted in Figure 4B, DOK3 mRNA levels were significantly upregulated in WT mice after CCI surgery (2.1-fold; *p <* 0.01), and the DOK3^-/-^ mice were validated by manifesting little DOK3 mRNA expression. In Figure 4C–4E, WT and DOK3^-/-^ mice showed 2.2- and 1.47-fold increases in TNF-α mRNA expression, 1.81- and 1.37-fold increases in IL-1β mRNA, and 1.65- and 1.64-fold increases in IL-6 mRNA expression compared with sham controls, respectively. In addition, except for IL-6, there were significant differences between genotypes. Moreover, CCI-induced upregulation of GPR84 and CD11B mRNA levels (but not of Arg1 levels) was dramatically inhibited in DOK3^-/-^ mice (Supplementary Figure 3B–3D). As shown in Figure 4F–4K, immunofluorescence (IFC) analysis indicated that the levels of Iba-1, p-p38, and GPR84 were increased in WT and DOK3^-/-^ mice after CCI; but in DOK3^-/-^ mice, they were significantly suppressed before and after surgery.

These *in vivo* results demonstrated that DOK3^-/-^ mice had a weakened state of CCI-induced inflammatory cytokine activity, which was significantly lower than in WT mice after CCI induction. Therefore, we posit that DOK3 plays a positive role in modulating inflammatory actions and microglial activation in response to peripheral neuropathy.

### GPR84 activation-induced mechanical allodynia and inflammatory responses are compromised in DOK3^-/-^ mice

**Figure 5:**
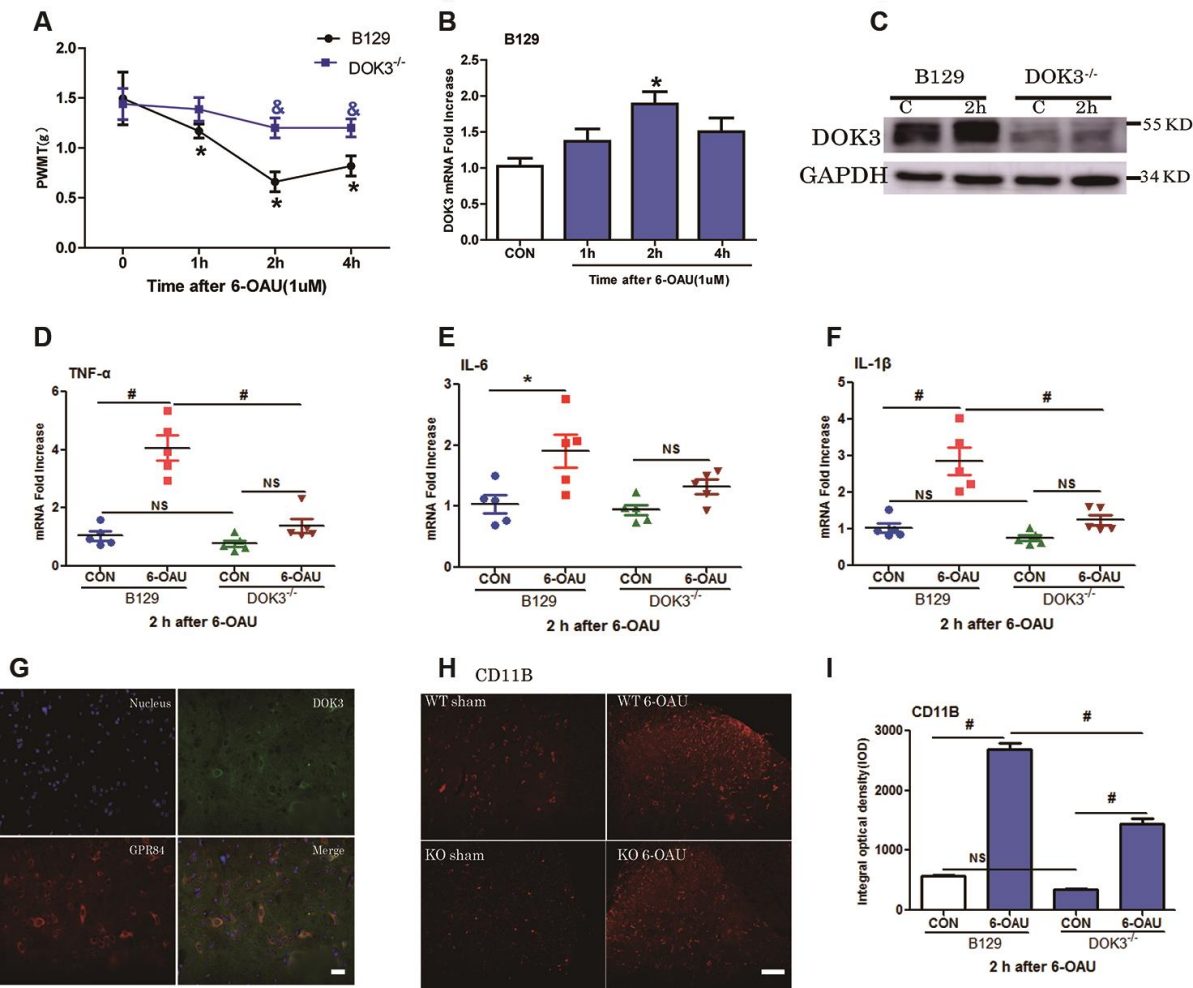
**GPR84 activation-induced mechanical allodynia and inflammatory responses are compromised in DOK3^-/-^ mice.** B129 and DOK3^-/-^ mice were treated with 6-OAU (1 μM) for 0, 1, 2, and 4 h via intrathecal injection. (**A**) GPR84 activation-induced mechanical allodynia was determined by calculating PWMT at 0, 1, 2, and 4 h after intrathecal injection. N=8-10, data are presented as means ± SEM. *p < 0.05 vs. control (CON) or 0 h; & *p <* 0.05 vs. WT mice at the same time after intrathecal injection. (**B**) DOK3 mRNA levels in mouse lumbar spinal cord were determined by RT-qPCR. (**C**) DOK3 protein levels were determined by western blotting 2 h after 6-OAU injection (1 μM). (**D**–**F**) Lumbar spinal cords were harvested from mice to determine the levels of mRNAs for TNF-α (**D**), IL-6 (**E**), and IL-1β (**F**) 2 h after 6-OAU injection (1 μM). (**G**) B129 mice were treated with 6-OAU (1 μM) for 2 h. The co-localization of GPR84 and DOK3 proteins was assessed using immunofluorescence analysis in mice. Scale bar, 20 μm. (**H**) The expression of CD11B proteins in lumbar spinal cord was assayed by immunofluorescence analysis and quantified (**I**). N=8-10, data are presented as means ± SEM. *p < 0.05, #*p <* 0.01 for comparisons between the 2 groups connected by the horizontal line; NS, not significant; scale bar, 50 μm.

As a previous study showed that GPR84 played an important role in neuropathic pain [23], we aimed to further determine the effect of DOK3 on GPR84-induced inflammatory responses in mice via intrathecal injection of a GPR84 agonist (6-OAU, 1 μM). As shown in Figure 5A, after 6-OAU treatment, mechanical pain thresholds of both WT and DOK3^-/-^ mice were reduced rapidly; however, there were significant differences between genotypes at 2 and 4 h. As expected, the levels of DOK3 mRNA were notably increased at 2 h, and this was further confirmed by western blot analysis in mouse spinal cord (Figure 5B, 5C and Supplementary Figure 4A). In addition, after 6-OAU treatment, WT and DOK3^-/-^ mice showed 2.8- and 1.65-fold increases in IL-1β, 4.0- and 1.79-fold increases in TNF-α, and 1.9- and 1.4-fold increases in IL-6 mRNA expression in spinal cords compared with sham controls, respectively (Figure 5D–5F).

As shown in Figure 5H, 5I, IFC analysis demonstrated that the levels of CD11B were increased in WT and DOK3^-/-^ mice after GPR84 activation; however, in DOK3^-/-^ mice, they were substantially inhibited before and after treatment, consistent with mRNA levels (Supplementary Figure 4B). Moreover, Supplementary Figure 4D shows that DOK3 was dominantly expressed in microglia in the mouse spinal cord, and we confirmed the co-localization of DOK3 and GPR84 (Figure 5G). Collectively, these results showed that 6-OAU-induced GPR84 activation promoted an inflammatory microglial phenotype in the spinal cord, and that the inflammatory conditions were largely dependent upon DOK3 function *in vivo*.

The ultimate goal of our research was to resolve clinical problems and effectively relieve pathologic pain. Since DOK3 was critical to neuropathic pain in mice, we hypothesized that supplementation with painkillers would prevent the development of inflammatory states by inhibiting DOK3.

### Administration of pregabalin attenuates neuropathic pain induced by enforced expression of DOK3 *in vivo*

A substantial number of clinical investigations have reported that pregabalin was effective in several models of neuropathic pain [34]. A single i.p. or oral treatment with pregabalin suppressed LPS-induced rectal hypersensitivity, as well as showed antinociceptive activity in a rat model of delayed visceral hyperalgesia induced by LPS administration. Moreover, other studies have indicated that pregabalin partially ameliorated damage caused by exposure to LPS in the hippocampus and cerebellum [35].

The ultimate goal of our research was to relieve pathologic pain and increase clinical relevance. Since DOK3 was critical to neuropathic pain in mice, we reasoned that supplementation of pregabalin might prevent the development of inflammatory states by inhibiting DOK3. Accordingly, we generated a plasmid harboring DOK3 cDNA or vector plasmid and introduced it into mice via intrathecal injection daily for 3 days. We then simultaneously administered a daily 14-day intragastric injection of pregabalin (Lyrica, Pfizer Manufacturing Deutschland GmbH).

**Figure 6:**
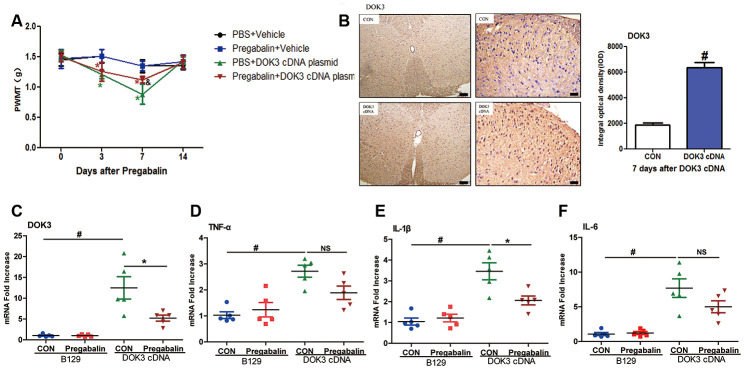
**Administration of pregabalin dramatically prevents mechanical allodynia and inflammatory responses induced by over-expression of DOK3.** B129 mice were treated with intrathecal injections of plasmid cDNA (5 μg) for 3 days and fed pregabalin (30 mg/kg/day) simultaneously for 2 weeks. (**A**) Paw-withdrawal mechanical threshold (PWMT) was evaluated on days 0, 3, 7, and 14. N=8-10, data are presented as means ± SEM. *p < 0.05 vs. baseline (day 0) & *p <* 0.05 vs. PBS plus plasmid cDNA on day 7. (**B**) Upregulation of DOK3 in lumbar spinal cords of mice after intrathecal injections of plasmid cDNA for 7 days was assessed by immunohistochemical analysis. Quantities were determined by calculating the integral optical density (IOD). N=8-10, #*p* < 0.01 vs. control (CON); scale bars, 100 μm and 20 μm. (**C**–**F**) mRNA levels for DOK3 (**C**), TNF-α (**D**), IL-1β (**E**), and IL-6 (**F**) in mouse lumbar spinal cord were determined by RT-qPCR on day 7. N=8-10; *p < 0.05; #*p <* 0.01; NS, not significant for comparisons between the 2 groups connected by the horizontal line.

As depicted in Figure 6A, intrathecal injection of the DOK3 cDNA plasmid into B129 mice effectively induced neuropathic pain from days 3 to 7, which recovered to basal levels on day 14. Administration of pregabalin was able to effectively relieve mechanical allodynia induced by overexpression of DOK3 on day 7 compared with mice carrying the DOK3 cDNA plasmid. Furthermore, the overexpression of DOK3 in spinal cord was confirmed by immunohistochemical and qPCR analyses (Figure 6B, 6C). We also found that DOK3 overexpression activated microglial cells in the spinal cord, but no activation of astrocytes was observed (Supplementary Figure 5C, 5D). Consistent with augmented DOK3 levels, the levels of TNF-α (2.72-fold), IL-1β (3.46-fold), and IL-6 (7.69-fold) were markedly increased compared to vector plasmid-treated mice (Figure 6D–6F); as were levels of CD11B (Supplementary Figure 5A). More importantly, our results showed that pregabalin significantly decreased DOK3 mRNA expression (58%, *p <* 0.05), that the inflammatory marker IL-1β was decreased by 41% (*p <* 0.05), that TNF-α decreased by 33% (NS), and that IL-6 decreased by 35% (NS) compared with plasmid cDNA-treated mice. We then further confirmed the effect of pregabalin on DOK3 in BV2 microglia. As shown in Supplementary Figure 6, exposure to pregabalin caused an effective inhibition of the LPS-induced increase in DOK3 levels. However, pregabalin had no effect on basal expression of DOK3 in BV2 microglia. In summary, these data provided evidence that pregabalin relieved neuropathic pain via inhibition of DOK3 expression in mice. Thus, targeting the DOK3 gene may in the future constitute a gene-therapy approach to alleviating diseases and injury-induced neuropathic pain in the spinal cord.

## DISCUSSION

In the present study, we identified a novel role for DOK3 protein in activated microglia-induced neuropathic pain as it induced immune responses and neuropathic pain by a physical interaction with GPR84. In microglial cells, knockdown of DOK3 markedly reduced the levels of inflammatory factors; and *in vivo*, CCI-induced neuropathic pain was compromised in DOK3^-/-^ mice. In addition, administration of pregabalin was effective in preventing DOK3 upregulation and DOK3 overexpression-induced neuropathic pain in mice. Therefore**,** our findings not only broadened the biologic role of DOK3 in chronic compression pain but also provided additional clinical relevance.

Recent studies have shown that DOK3—highly expressed in B cells and macrophages—binds the inhibitory molecules SHIP, Grb2, and Csk to inhibit immunoreceptor signaling of NF-кB, p38MAPK, JNK, nuclear factor of activated T-cells (NFAT), and proinflammatory cytokine release—as well as B cell hyper-proliferation [14, 15, 29, 36, 37]. Intriguingly, DOK3 also exhibits a proinflammatory role when responding to several stimulatory factors, e.g., DOK3 induced IFN-β production and TBK1 and TRAF3 activation [22]. We speculate that the functions of DOK3 proteins depend not only on the different molecules they bind but also upon the different periods of time and physiologic/pathologic situations to which they are exposed. Although a role for DOK3 was previously reported in B cells and macrophages, our study is the first to identify a significant role for DOK3 in microglial cells.

The major finding of the present study was that DOK3 in microglial cells was crucial for CCI-induced pain. A growing body of evidence has shown that activated microglial cells are involved in host defense, production of inflammatory cytokines in response to injury, and infection and pain; and our study also provided evidence to support these data. Spinal cord injury (SCI) resulted in motor impairment and chronic central pain, and intrathecal infusions of minocycline (a microglial inhibitor) rapidly attenuated hyperalgesia and hyperresponsiveness [9]. Neurons and glial cells interact in a complex manner, mediating numerous pathologic processes in the central nervous system [11]. In addition, spinal microglia directly influence the occurrence and maintenance of neuropathic pain via a series of signal-transduction pathways, including those involving purinergic P2X4 receptors, CC chemokine receptors, and mitogen-activated protein kinases [13]. Previous studies showed that peripheral nerve injury (PNI) upregulated MafB expression in spinal microglia, with the transcription factor MafB activating microglia to induce neuropathic pain [38]. In addition, inhibiting the p38 MAPK-signaling pathways in spinal microglia attenuated neuropathic pain in rats [39, 40]. Microglia are also important in stress phenomena, depressive disorders, and pain-associated brain response [41]. Therefore, inhibition of microglial activity and subsequent modulation of neuroinflammation effectively relieve neuropathic pain in animals.

It was reported in a plethora of studies that GPR84 contributes to the production of inflammatory mediators and the maintenance of neuropathic pain in microglia. For example, 6-OAU (a surrogate agonist of GPR84) induced chemotaxis of macrophages and increased the production of IL-8 and TNF-α [25]; and GPR84 knock-out (KO) mice did not exhibit mechanical or thermal hypersensitivity after partial sciatic nerve ligation (PNL) [23]. Furthermore, as a proinflammatory receptor, GPR84 was also involved in inflammatory and immune responses. GPR84 was highly expressed in microglial cells in mice that manifested experimental autoimmune encephalomyelitis (EAE) and endotoxemia [24]. Highly expressed GPR84 in zebrafish markedly increased the number of phagocytic macrophages, which proved that GPR84 exerts direct effects on immune reactions [42]. Additionally, lipopolysaccharide induced an upregulation of GPR84 expression in activated macrophages and monocytes [43]. Investigators have concluded that GPR84 exerts a beneficial action in Alzheimer’s disease by promoting microglial recruitment and preventing cognitive decline [44]. Our results strongly support the hypothesis that DOK3 functions by generating a signaling complex with GPR84 in microglial cells. However, due to the lack of relevant bioinformatics analyses, the specific binding sites between DOK3 and GPR84 molecules require further elucidation. We also cannot exclude the possibility that this signaling complex includes other unknown molecules, and this prospect will direct us in our future work.

As a commonly used clinical analgesic drug for neuropathic pain, pregabalin [S-(1)-3-isobutylgaba] is a novel agent that functions in analgesic, anticonvulsant, and anxiolytic activities; and can block voltage-dependent calcium channels and reduce the release of neurotransmitters. Our results showed that pregabalin significantly decreased DOK3 mRNA expression and the induction by upregulated DOK3 of the inflammatory markers IL-1β, TNF-α, and IL-6. We thus speculated that pregabalin exerted a direct or indirect regulatory effect on DOK3 function. By attenuating DOK3 protein levels, pregabalin reduced the biosynthesis of inflammatory markers in the spinal cord and reduced neuropathic pain. In the present study, we broadened the potential application of pregabalin in the improvement of upregulated DOK3-induced neuropathic pain in mice. This suggested to us that DOK3 may be a potential key factor in pregabalin targeting, but additional evidence related to its clinical effectiveness is still needed.

We realize that our study possesses some limitations. We used male mice in our experiments, so additional studies will need to be performed to explore the effects of sex differences on pain behavior so as to make our investigation more comprehensive and authoritative.

We herein summarize our results as follows. First, knockdown of DOK3 contributed to reduced levels of inflammatory factors in microglia; second, down-regulation of DOK3 weakened the ability of microglia to release inflammatory mediators under GPR84-induced pathologic conditions; and third, CCI-induced neuropathic pain hypersensitivity was attenuated in DOK3^-/-^ mice. In conclusion, DOK3 knockdown significantly reduced the allodynia caused by chronic compression injury. Another important finding was supported by several results. First, co-localization of DOK3 and GPR84 was detectable in microglia by using IFC analysis; second, purified recombinant DOK3 proteins bound to GPR84 proteins directly *in vitro*, suggesting a physical association between DOK3 and GPR84 *in vivo*; and third, GPR84 activation-induced mechanical allodynia was compromised in DOK3^-/-^ mice.

Collectively, our results suggest that DOK3 plays a significant role in neuropathic pain. We therefore propose that CCI-induced injury activates GPR84 and that GPR84 subsequently recruits DOK3 to mediate the synthesis and release of downstream inflammatory factors, leading to mechanical allodynia and the formation of central sensitization. We postulate that the characterization of specific immune cell expression and proinflammatory profiles will constitute DOK3 as a novel target in the treatment of chronic pain and central sensitization.

## MATERIALS AND METHODS

### Animals and surgery

Adult (8-week-old) male B129 mice were purchased from Beijing Vital River Laboratory Animal Technology Co., Ltd. The DOK3 knockout (KO) mice (on a B129 background) were obtained from The Key Laboratory of Cardiovascular Remodeling and Function Research, Cheeloo College of Medicine, Shandong University. We used the fewest number of mice statistically possible for each experiment. The animals were housed on a 12-h light-12-h dark cycle at a room temperature of 22 ± 2° C with free access to food and water. Our study was performed in accordance with the recommendations in the Guide for the Care and Use of Laboratory Animals of the National Institutes of Health, USA. This animal protocol was reviewed and approved by the Animal Care and Use Committee of Shandong University. Chronic constriction injury of the sciatic nerve (CCI) was performed according to a previously published protocol [45].

### Intrathecal injection and behavioral testing

In brief, we injected into the subarachnoid space between vertebrae L5 and L6, which was site- validated by the appearance of a tail flick. The paw-withdrawal mechanical threshold (PWMT) was evaluated on 3–5 separate days before surgery and on days 3, 7, 14, and 21 after surgery by using a BME-404 Mechanical Analgesia Tester (Chinese Academy of Medical Sciences, CAMS, Beijing, China) [46]. Paw withdrawal latency (PWL) to heat in mice was determined with a BME-410C thermal analgesia tester (CAMS). All of the procedures were performed by the same investigator. Specific experimental steps are described in the Supplementary Materials.

### Microglial cell culture and lentiviral infection

BV2 microglia were purchased from Procell Life Science and Technology Co., Ltd., and maintained in MEM supplemented with 10% endotoxin-free fetal bovine serum infected with lentiviruses according to the manufacturer’s instructions. DOK3 shRNA lentiviral particles and negative control shRNA were synthesized by Shanghai Genechem Co., Ltd. For transfection, shRNA lentiviral particles were used according to the manufacturer’s instructions [47].

### Cell immunofluorescence (IFC) and immunohistochemistry

Cultured microglia and tissues were dehydrated and embedded in paraffin. After antigen retrieval and washing, the sections were heated and deparaffinized, and incubated with primary antibody. We examined the cells under a SLM 510 laser-scanning confocal microscope (Carl Zeiss Meditec, Jena, Germany). Detailed experimental methods for cell immunofluorescence (IFC) and immunohistochemistry are described in the Supplementary Materials.

### Western blotting and PCR analyses

Cultured cells and lumbar spinal cords were collected after treatments. We separated protein samples on SDS-PAGE gels and transferred them to polyvinylidene difluoride membranes. The blots were incubated with antibodies against DOK3 (1:500, Abcam), GPR84 (1:500, Biorbyt), p-p38 (1:500, Santa Cruz), Iba-1 (1:800, Abcam), or GST (1:800, Abcam). For the protein loading control, we incubated the blots with GAPDH antibody (1:1000, Abcam). We also used a Na^+^/K^+^-ATPase antibody (1:1000, Abcam) as a control for membrane proteins. Blots were visualized with the enhanced chemiluminescence system (Millipore, Beijing, China), and intensities of the selected bands were analyzed using Image J software (NIH, Bethesda, MD, USA).

PCR analysis was performed according to the manufacturer’s protocol (Toyobo, Japan), and GAPDH was used as an endogenous control to normalize differences in mRNA detection. Melting curves were created upon completion of the cycles to confirm that nonspecific products were absent. We quantified mRNA levels by normalizing Ct (cycle threshold) values with GAPDH Ct, and analyzed them using the 2^-ΔΔCT^ method. The primer sequences used are described in the Supplementary Materials.

**Co-immunoprecipitation and GST Pull-down Assays**

We prepared cell lysates with ice-cold RIPA buffer as previously described [48]. Immunoprecipitation was performed by incubating the precleared cellular lysates with primary antibody (anti-DOK3 antibodies, Abcam) on ice for 1 h, and we added protein A or protein G agarose beads (Santa Cruz) at 4° C for 1 h with rotation. The beads were then washed, and the immunoprecipitation complexes were dissolved in SDS-PAGE sample buffer before being resolved by SDS-PAGE for western blotting. We transferred proteins onto polyvinylidene difluoride membranes, incubated them with primary antibody (anti-GPR84 antibody, Santa Cruz), and visualized them with an enhanced chemiluminescence system (Pierce). Recombinant GST alone and the recombinant GST-DOK3 fusion protein (Abnova, Taiwan) were combined with affinity glutathione-agarose beads (Santa Cruz), as described previously [31]. The protein-bead complexes (15–20 mg of proteins) were incubated with microglial cell lysates (~1 mg) at 4° C for 2 h with rotation. The beads were then washed 3 times with RIPA buffer and dissolved in SDS-PAGE sample buffer for analysis by western blotting.

### Statistical analysis

All of the numerical data are shown as means ± SEM. We detected statistical significance between groups using 1-way ANOVA followed by Bonferroni post-hoc testing and Student’s t-test, except for those behavioral measurement data, which were analyzed with repeated-measures ANOVA. Normally distributed data were validated with the Kolmogorov-Smirnov analysis before performing statistical comparisons. We conducted all of the statistical analyses using IBM SPSS statistics 20.0 (IBM, Armonk, NY) and GraphPad Prism software version 5 (GraphPad Software, Inc, San Diego, CA). *p <* 0.05 was considered to be statistically significant.

## Supplementary Material

Supplementary Materials

Supplementary Figures
