# Supplementary material for: DOK3 is involved in microglial cell activation in neuropathic pain by interacting with GPR84
Source: Aging (Albany NY). 2020 Dec 3;13(1):389–410. doi: 10.18632/aging.202144 (PMC7835011; doi:10.18632/aging.202144)
Supplement: Supplementary Materials [file aging-13-202144-s001.pdf]

MATERIALS AND METHODS

Materials

Lipopolysaccharide (LPS) from *Escherichia coli* 0111:B4 was purchased from Sigma (St. Louis, MO), dissolved in pyrogen-free saline, and incubated *in vitro* at a dose of 1 μM. 6-n-Octylaminouracil (6-OAU) was purchased from MedChemExpress (Shanghai, China) and dissolved in DMSO. Control groups were treated with DMSO only. The concentration used were 1 μM, 10 μM, or 100 μM *in vitro*; and 1 μM *in vivo*, respectively. The cDNA plasmid encoding DOK3 proteins and non-targeting-scrambled control vectors were synthesized by Biosune (Shanghai, China). DOK3 shRNA lentiviral particles (NM_013739: 5'-GACAAGGGTGTGTTCTCGTTT-3') and negative control shRNA (5'-TTCTCCGAACGTGTCACGT-3') were synthesized by Shanghai Genechem Co., Ltd.

For mRNA detection, the total RNA of cultured cells and lumbar spinal cord were extracted with Trizol reagent (Invitrogen, Carlsbad, CA). Fragments were then amplified with appropriate primers, using the following primers : GPR84 (forward, 5'-CGT GGC AGT GGC TGT GAC AG-3'; reverse, 5'-TGA GGT TGG CAA TGA GCA GGT TG-3'); DOK3 (forward, 5'-AGA AAG CTG CCT CTA ACT GAT C-3'; reverse, 5'-ATG CAC ACG TTC TCA TAG AGA T-3'); GAPDH (forward, 5'-AGC CCA GAA CAT CAT CCC TG-3'; reverse, 5'-CAC CAC CTT CTT GAT GTC ATC-3'); TNF-α (forward, 5'-CAC CAC GCT CTT CTG TCT-3'; reverse, 5'-GGG CTT GTC ACT CGA GTT-3'); IL-1β (forward, 5'-TGC ACT GCA GGC TTC GAG AT-3', reverse, 5'-CCA AGG CCA CAG GGA TTT TG-3'); IL-6 (forward, 5'-CTC CCA ACA GAC CTG TCT ATA C-3'; reverse, 5'-CCA TTG CAC AAC TCT TTT CTC A-3'); CD11B (forward, 5'-TCC CAC AGC CAG CGG ATC ATA G-3'; reverse, 5'-CCA ACA GCC AGG TCC ATC AAG C-3'); Arg1 (forward, 5'-TGT CCC TAA TGA CAG CTC CTT-3'; reverse, 5'-GCA TCC ACC CAA ATG ACA CAT -3'); CD206 (forward, 5'-AGG CTG ATT ACG AGC AGT GG-3'; reverse,5'-CCA TCA CTC CAG GTG AAC CC-3'); P38 (forward, 5'-GCG GAC CTG AAC AAC ATC GT-3'; reverse, 5'-CTG GGC TTT AGG TCC CTG TGA-3'); iNOS (forward, 5'-CCT GCT TTG TGC GAA GTG TC-3'; reverse, 5'-CCC AAA CAC CAA GCT CAT GC-3'); and CD68 (forward, 5'-ACT TCG GGC CAT GTT TCT CT-3'; reverse, 5'-GGG GCT GGT AGG TTG ATT GT-3'). The primers were synthesized by Biosune (Shanghai, China).

CCI model

Our model of chronic constriction injury of sciatic nerve (CCI) was constructed as follows. Mice were anesthetized with sodium pentobarbital (50 mg/kg body weight, i.p.), and the skin on the left mid-thigh was shaved and incised (5-8 mm). The proximal and distal parts of the biceps femoris muscle were separated to expose the common sciatic nerve, and the nerve was dissected away from surrounding tissues. Three ligatures of 6-0 silk were tied around the nerve just proximal to the trifurcation such that 1/3 to 1/2 of the nerve was held within the ligature. The desired degree of constriction retarded—but did not arrest—circulation through the superficial epineurial vasculature; and sometimes produced a small, brief twitch in the muscle surrounding the exposure. For the sham-operated controls, the mice underwent the same procedure without constriction of the nerve. The incision was closed in layers and a single dose of buprenorphine (0.05 mg/kg, s.c.) was administered. The mice were placed in clean cages and carefully monitored until fully awake [1–3].

Intrathecal injection and drug administration

Before intrathecal injection, the constructed cDNA plasmid encoding DOK3 protein, and non-targeting-scrambled control vector were mixed with Lipofectamine 2000 reagent (Invitrogen, Carlsbad, CA). We mixed 1 μg of plasmid with 1 μL of reagent for *in vivo* experiments, and then allowed complex formation to occur for 10 min. Intrathecal injection was performed on the anesthetized mice as follows [4, 5]. In brief, an injection (5 μL) into the subarachnoid space between the L5 and the L6 vertebrae was performed using a 27-gauge needle; and entry of the needle was confirmed by the presence of a tail flick. Within each individual study, 1 group of 6 mice was given intrathecal injections of plasmid cDNA daily for 3 days; and simultaneously, a daily intragastric injection of pregabalin [*S*-(+)-3-isobutylgaba] in capsules for 10 days. Capsules were dissolved in sterile NaCl (0.9% isotonic saline) immediately before use. For the sham-gavaged controls, mice underwent the same procedure with only sterile NaCl. Dosing volumes were 30 mg/kg body weight, in accordance with a previous study [6].

Behavioral testing

For behavioral analysis, animals were habituated to the testing environment daily for at least 2 days before baseline testing. They were then placed in boxes on an elevated metal mesh floor and allowed to habituate for 30 min. During testing, a probe was pressed against the lateral plantar surface of the hind paws with sufficient force to evaluate the paw-withdrawal mechanical

threshold (PWMT); a positive response was noted when the paw immediately withdrew. The procedure was repeated 5 times at least 5 min apart, and the average value was used as a variable [7]. Walk-gait pattern was assessed as an index of motor function. Only animals that indicated a normal gait, without any foot deformities, were used for the following experimental procedures. Paw withdrawal latency (PWL) to heat in mice was determined with the BME-410C thermal analgesia tester (CAMS). Before testing, the mice were randomly placed in testing cubicles and acclimated for 30 minutes, and then the thermal analgesia tester was calibrated to produce a paw-withdrawal latency of 8–12 s in naive control mice. Radiant light was directed onto the center of the hind paw through the glass plate, and when the mouse lifted or licked his hind paw, PWL was measured automatically. A 25-s cutoff period was used to protect hind-paw tissue. Each hind paw was stimulated 3 times with a 5-min interval, and the mean withdrawal latency was recorded.

Microglial cell culture and treatment

BV2 microglia were maintained in MEM supplemented with 10% endotoxin-free fetal bovine serum, and incubated at 37° C in a humidified atmosphere of 5% CO_2 and 95% air. We grew cells to 70%–80% confluency before being treated with different agents. DOK3 shRNA lentiviral particles and negative control shRNA were synthesized by Shanghai Genechem Co., Ltd; and shRNA lentiviral particles were used according to the manufacturer's instructions for transfection. Briefly, microglial cells were cultured to 50% confluency. The cells were infected with lentiviruses according to the recommended multiplicity of infection at approximately 10, and after 72 h, lentivirus infection-stable clones expressing the DOK3-specific shRNA were selected with puromycin (5 μg/ml).

Pregabalin powder was dissolved in phosphate-buffered saline (PBS). BV2 microglia were pre-treated with pregabalin at a concentration of 10 μg/ml for 2 hour, and then cells were incubated with LPS (1 μM) for 12 hours. The control groups were incubated with PBS without pregabalin or LPS, or with neither. The concentration of pregabalin was chosen according to a previous report [8].

Cellular Immunofluorescence (IFC) and Immunohistochemistry

Prior to stimulation, cultured microglia grown on sterile-glass cover slips were rinsed with cold PBS and then fixed in 10% formalin/PBS for 10 min. We blocked cells with 5% BSA for 30 min. Double-staining for DOK3 and GPR84 was performed according to the manufacturer's protocol, as described previously[9]. We incubated cells with primary antibody (anti-DOK3 antibody, 1:100, Abcam, Cambridge, UK; or anti-GPR84 antibody, 1:100, Biorbyt) for 1 h at room temperature or overnight at 4° C. After washing, we incubated with fluorescence-conjugated secondary antibody (1:200; Genecopoeia, USA) for 45 min. The nuclei were stained with 4′,6-diamidino-2-phenylindole, and we examined the cells under a SLM 510 laser-scanning confocal microscope (Carl Zeiss Meditec, Jena, Germany).

After the behavioral and pain tests, mice were overdosed with sodium pentobarbital (50 mg/kg, i.p.) and perfused transcardially with 0.9% saline, followed by fixation with 4% paraformaldehyde in 0.1 M phosphate buffer. Lumbar spinal cords were dissected and assayed for DOK3, GPR84, Iba-1, p-p38, and CD11B according to previous studies [7]. Tissues were fixed in the same fixative overnight at 4° C, and then dehydrated and embedded in paraffin. A series of 4-μm paraffin sections were cut using a rotary microtome, and we heated the sections at 65° C for at least 2 h and then deparaffinized them. Antigen retrieval was accomplished with citrate buffer in a microwave oven at 92° C–98° C for 15 min. The sections were incubated separately with primary antibody (anti-Iba-1 antibody, 1:200, Abcam; anti-P-p38 antibody, 1:100, Santa Cruz; anti-CD11B antibody, 1:100, Abcam; or anti-GPR84 antibody, 1:100, Biorbyt) at 4° C overnight. The sections were then incubated using a specific secondary antibody for 2 h at room temperature, and DAB substrate solution and hematoxylin were used to develop color. As for immunofluorescence, we incubated sections with fluorescence-conjugated secondary antibody for 2 h, and nuclei were stained with 4′,6-diamidino-2-phenylindole. Labeled sections were examined under a Leica Quantimet 550 DMRXA automated research microscope (GER) and analyzed using IPP.6.

REFERENCES

1. Bennett GJ, Xie YK. A peripheral mononeuropathy in rat that produces disorders of pain sensation like those seen in man. Pain. 1988; 33:87–107. https://doi.org/10.1016/0304-3959(88)90209-6 PMID:2837713

2. Nakamura Y, Morioka N, Abe H, Zhang FF, Hisaoka-Nakashima K, Liu K, Nishibori M, Nakata Y. Neuropathic pain in rats with a partial sciatic nerve ligation is alleviated by intravenous injection of monoclonal antibody to high mobility group box-1. PLoS One. 2013; 8:e73640. https://doi.org/10.1371/journal.pone.0073640 PMID:23991202

3. Seltzer Z, Dubner R, Shir Y. A novel behavioral model of neuropathic pain disorders produced in rats by partial sciatic nerve injury. Pain. 1990; 43:205–18. https://doi.org/10.1016/0304-3959(90)91074-s PMID:1982347

4. Hylden JL, Wilcox GL. Intrathecal morphine in mice: a new technique. Eur J Pharmacol. 1980; 67:313–16. https://doi.org/10.1016/0014-2999(80)90515-4 PMID:6893963

5. Nakamura Y, Morioka N, Zhang FF, Hisaoka-Nakashima K, Nakata Y. Downregulation of connexin36 in mouse spinal dorsal horn neurons leads to mechanical allodynia. J Neurosci Res. 2015; 93:584–91. https://doi.org/10.1002/jnr.23515 PMID:25400139

6. Eutamene H, Coelho AM, Theodorou V, Toulouse M, Chovet M, Doherty A, Fioramonti J, Bueno L. Antinociceptive effect of pregabalin in septic shock-induced rectal hypersensitivity in rats. J Pharmacol Exp Ther. 2000; 295:162–67. PMID:10991974

7. Qu YJ, Zhang X, Fan ZZ, Huai J, Teng YB, Zhang Y, Yue SW. Effect of TRPV4-p38 MAPK pathway on neuropathic pain in rats with chronic compression of the dorsal root ganglion. Biomed Res Int. 2016; 2016:6978923. https://doi.org/10.1155/2016/6978923 PMID:27366753

8. Abu-Rish EY, Mansour AT, Mansour HT, Dahabiyeh LA, Aleidi SM, Bustanji Y. Pregabalin inhibits in vivo and in vitro cytokine secretion and attenuates spleen inflammation in Lipopolysaccharide/Concanavalin A -induced murine models of inflammation. Sci Rep. 2020; 10:4007. https://doi.org/10.1038/s41598-020-61006-1 PMID:32132609

9. Yang XH, Li P, Yin YL, Tu JH, Dai W, Liu LY, Wang SX. Rosiglitazone via PPARγ-dependent suppression of oxidative stress attenuates endothelial dysfunction in rats fed homocysteine thiolactone. J Cell Mol Med. 2015; 19:826–35. https://doi.org/10.1111/jcmm.12510 PMID:25656735
