# Supplementary material for: DOK3 is involved in microglial cell activation in neuropathic pain by interacting with GPR84
Source: Aging (Albany NY). 2020 Dec 3;13(1):389–410. doi: 10.18632/aging.202144 (PMC7835011; doi:10.18632/aging.202144)
Supplement: Supplementary Figures [file aging-13-202144-s002.pdf]

SUPPLEMENTARY FIGURES

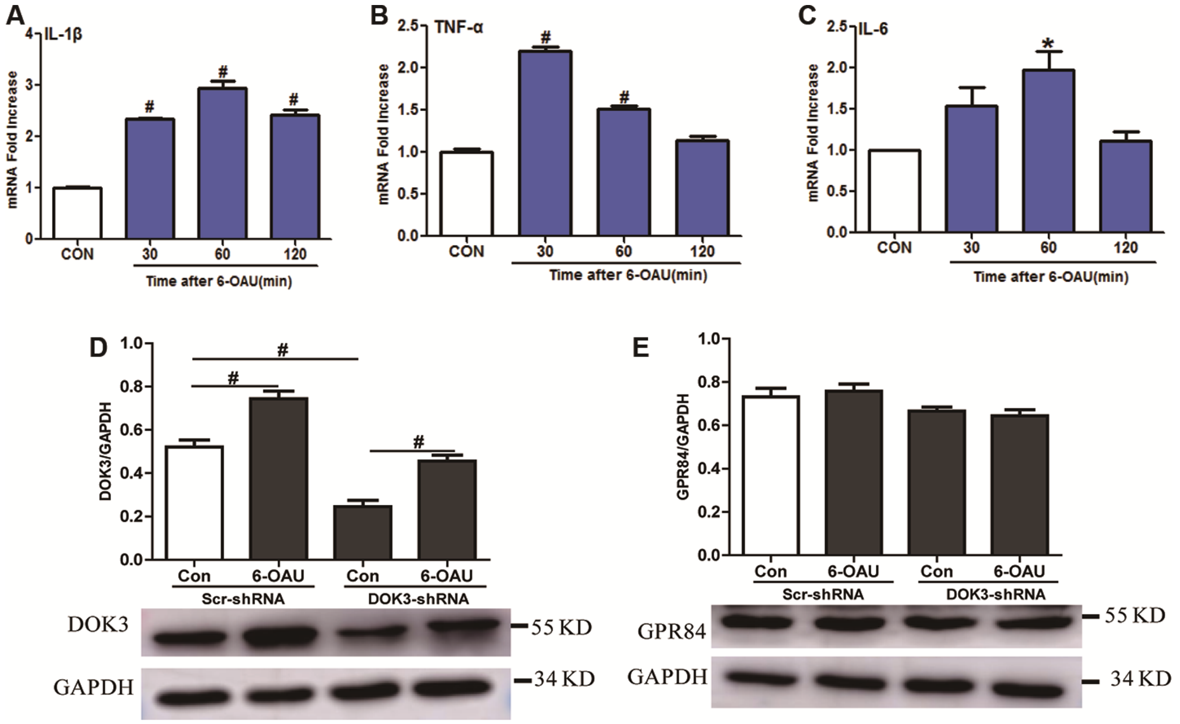

Supplementary Figure 1. GPR84 activation induces inflammatory responses in microglia. (**A**–**C**) Microglia were incubated with 6-n-octylaminouracil (6-OAU) for 30, 60, and 120 min (1 μM). IL-1β (**A**), TNF-α (**B**), and IL-6 (**C**) mRNA levels were determined by RT-qPCR. (**D**, **E**) Lentivirus-infected microglia were exposed to 6-OAU (1 μM, 60 min). Protein levels for DOK3 (**D**) and GPR84 (**E**) were determined by western blotting analysis. N=3 per group, data are presented as means ± SEM. **p* < 0.05, #*p* < 0.01, NS, not significant vs. control (CON) or between the 2 groups connected by the horizontal line.

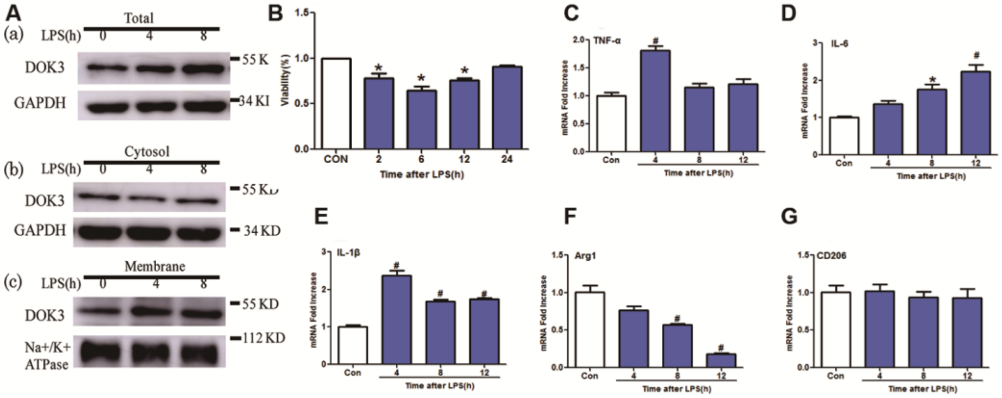

Supplementary Figure 2. LPS induces inflammatory responses and translocation of DOK3 from cytosol to the plasma membrane in microglia. (**A**) Microglia were pretreated with LPS (1 μM) for 0, 4, or 8 h; and total cell lysates were assayed by using a membrane and cytoplasmic protein extraction kit and western blotting. (**B**) Microglia were pretreated with LPS (1 μM) for 0, 2, 6, 12, and 24 h; and cellular viability was determined using a cell counting kit-8 (CCK-8). (**C**–**G**) Microglia were pretreated with LPS (1 μM) for 0, 4, 8, or 12 h; and mRNA levels for TNF-α (**C**), IL-6 (**D**), IL-1β (**E**), Arg1 (**F**), and CD206 (**G**) were determined by RT-qPCR. N=3 per group, data are presented as means ± SEM. **p* < 0.05, #*p* < 0.01 vs. control (CON).

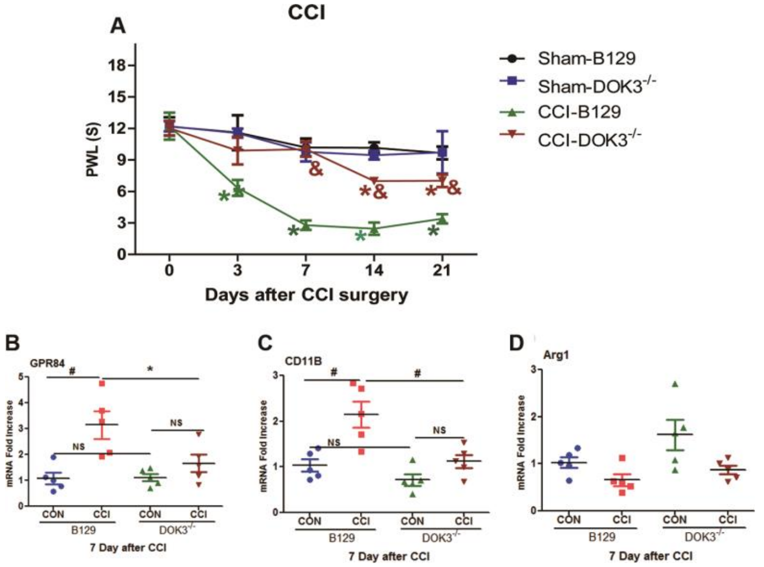

Supplementary Figure 3. CCI-induced up-regulation of thermal allodynia partially inhibits GPR84, CD11B, and Arg1 in DOK3$^{-/-}$ mice. B129 and DOK3$^{-/-}$ mice underwent surgery by chronic constriction of the sciatic nerve to establish the CCI model. (**A**) CCI-induced thermal allodynia was determined by calculating paw withdrawal latency (PWL) to heat on days 0, 3, 7, 14, and 21 after CCI surgery. N=8-10, data are presented as means ± SEM. *p < 0.05 vs. sham group, &p < 0.05 vs. WT plus CCI at the same time. (**B**–**D**) Homogenates of lumbar spinal cords in mice were used to determine the levels of mRNA for GPR84 (**B**), CD11B (**C**), and Arg1 (**D**) on day 7 after CCI. N=8-10; *p < 0.05, #p < 0.01; NS, not significant for comparisons between the 2 groups connected by the horizontal line.

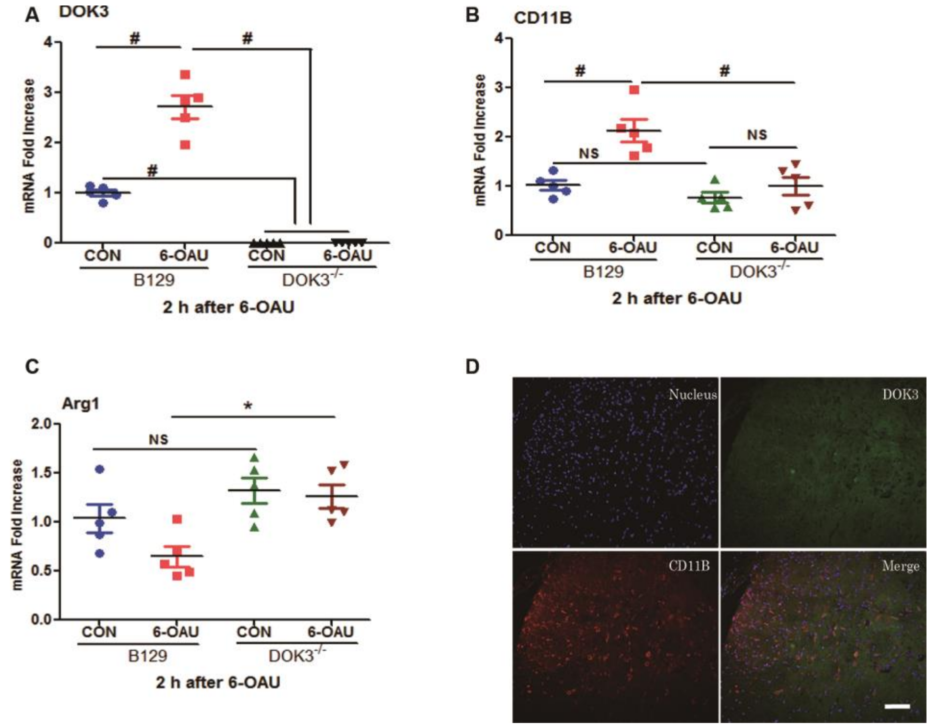

Supplementary Figure 4. GPR84 activation-induced inflammatory responses are compromised in DOK3$^{-/-}$ mice. (**A**–**C**) B129 and DOK3$^{-/-}$ mice were treated with 6-OAU (1 μM) for 2 h via intrathecal injection, and mouse lumbar spinal cords were harvested to determine the mRNA levels for DOK3 (**A**), CD11B (**B**), and Arg1 (**C**). N=8-10, data are presented as means ± SEM. *p < 0.05, #p < 0.01 for comparisons between the 2 groups connected by the horizontal line; NS, not significant. (**D**) B129 mice were treated with 6-OAU (1 μM) for 2 h. The co-localization of DOK3 and CD11B proteins was evaluated by immunofluorescence analysis of mouse lumbar spinal cords. Scale bar, 50 μm.

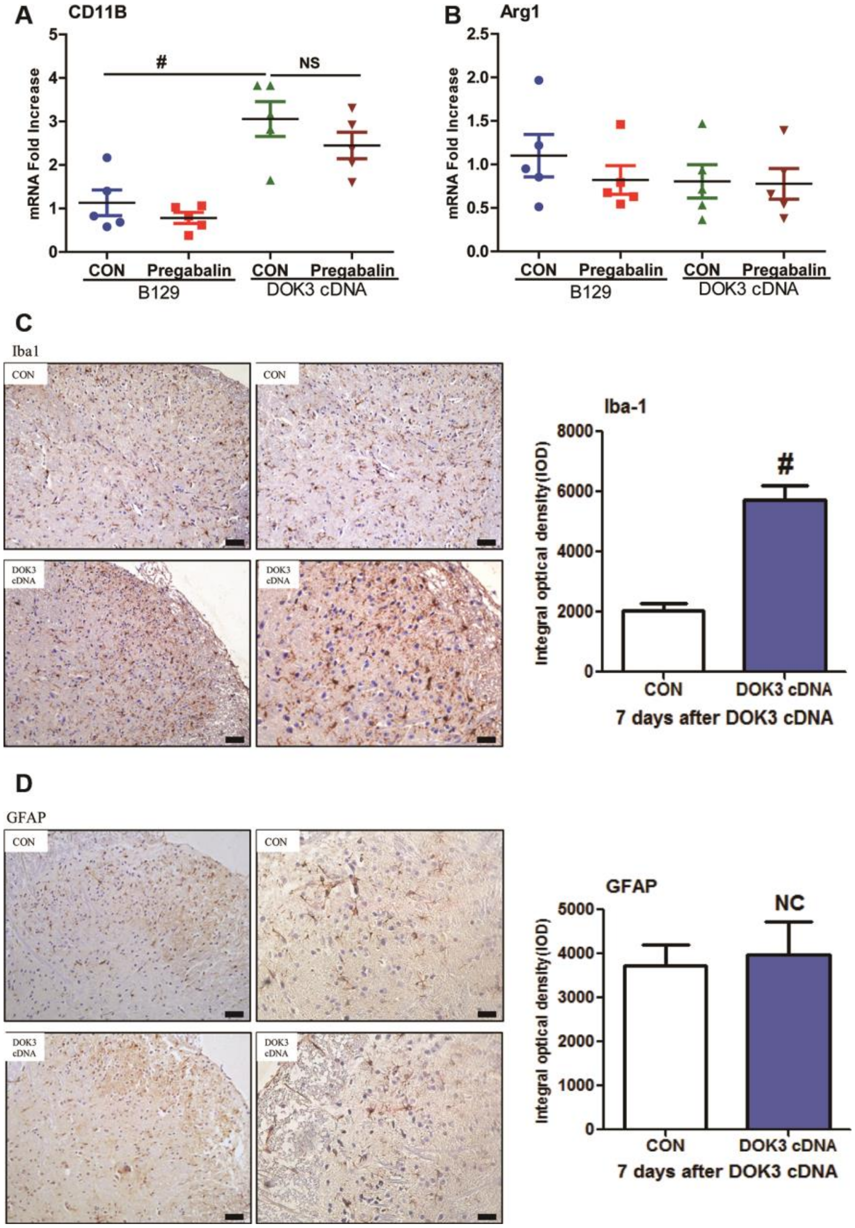

Supplementary Figure 5. The effect of pregabalin administration on CD11B and Arg1, and on activation of spinal cord cells. (**A**, **B**) Mice were treated with intrathecal injections of plasmid cDNA (5 µg) for 3 days and fed with pregabalin simultaneously (30 mg/kg/day) for 2 weeks. CD11B (**A**) and Arg1 (**B**) mRNA levels in mouse lumbar spinal cords were determined by RT-qPCR on day 7. N=8-10; **p* < 0.05, #*p* < 0.01; NS, not significant for comparisons between the 2 groups connected by the horizontal line. (**C**, **D**) Expression of Iba-1(C) and GFAP (D) in lumbar spinal cords of mice after intrathecal injections of plasmid cDNA for 7 days were assayed by immunohistochemical analysis. Quantities were determined by calculating the integral optical density (IOD). N=8-10; #*p* < 0.01 vs. control (CON); NS, not significant; scale bars, 50 µm and 20 µm.

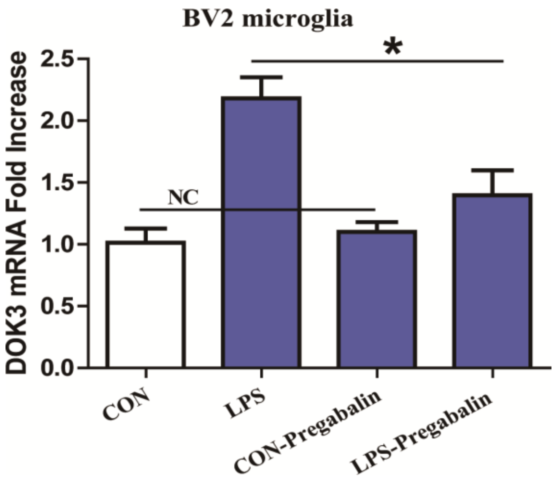

Supplementary Figure 6. Effect of pregabalin administration on DOK3 in BV2 microglia. BV2 microglia were pretreated with pregabalin for 2 h, followed by incubation with LPS (1 μM) for 12 hours; and cells were collected to determine DOK3 mRNA levels. N=3 per group, data are presented as means ± SEM. *$p < 0.05$; NS, not significant for comparisons between the 2 groups connected by the horizontal line.
